# CHEMICAL LEUCODERMA: INDIAN SCENARIO, PROGNOSIS, AND TREATMENT

**DOI:** 10.4103/0019-5154.70674

**Published:** 2010

**Authors:** A K Bajaj, Abir Saraswat, P K Srivastav

**Affiliations:** *From the Bajaj Skin Clinic, Allahabad, India*; 1 *From the Indushree Skin Clinic, Lucknow, India*

**Keywords:** *Chemical leucoderma*, *contact depigmentation*, *bindi depigmentation*, *alta*, *azo dyes*, *paraphenylenediamine*, *hair dye*, *black henna*, *wallet depigmentation*, *footwear*

## Abstract

Chemical leucoderma is an industrial disorder in developed countries and the common causative chemicals are phenols and catechols. Due to stringent controls and preventive measures the incidence has come down. In the recent past various chemicals in consumer products have also been documented to produce depigmentation. In India due to lax quality control measures chemical leucoderma due to consumer items is not uncommon.The various consumer items documented to cause contact depigmentation are sticker bindis, rain shoes, plastic chappals, hair dye/ black henna(kali mehndi), alta, wallets and even mobile plastic covers.

## Introduction

The color of skin, more so the patchy lack of it, has tremendous psychosocial significance in our society. Some of the most distressed patients seen by any Indian dermatologist are those who have depigmented skin lesions. Patients with depigmentation are variously perceived as being unclean, contagious, or both, and these lesions are often mistaken for stigmata of leprosy or some hereditary diseases.

While the majority of patients with *de novo* depigmented lesions suffer from idiopathic vitiligo, some do have an identifiable culprit that causes melanocyte destruction. These cases occur due to direct skin exposure to some chemicals that are selectively toxic to melanocytes. This exposure can occur either in the workplace[1] or even in day-to-day life with common objects that come in contact with the skin.[2] However, chemical leucoderma is relatively uncommon whereas the chemicals implicated in it are widely used and commonly present in the environment. From this, it is evident that some individuals have inherently “fragile” melanocytes that are more susceptible to injury upon exposure to these chemicals. It has been shown both *in vitro* and *in vivo* that certain aliphatic and aromatic derivatives of phenols and catechols are directly toxic to melanocytes,[3] more so in genetically susceptible individuals.[4] Other chemicals that are known to cause chemical leucoderma are *p*-phenylenediamine (PPD), certain azo dyes, sulfhydryls, mercurials, arsenic, and several drugs of chemically related classes.[5]

From the consumer’s point of view, these chemicals are present in a variety of day-to-day products, such as lightening agents in bleaching creams (hydroquinone),[6] in common household adhesives (*p*-tertiary butylphenol, PTBP),[7] and as dyes in hair colors and black henna (PPD).[8] In addition, several traditional materials of cosmetic use in Asian, specifically Indian women are laced with these chemicals in their modern *avatars. Bindi*, which was traditionally made with mineral- or vegetable-based dyes, is now a synthetic fabric or plastic patch backed by an adhesive glue that contains *p*-tertiary butyl phenol. *Alta*, a traditional red dye used to color the feet, and hands of women now contain a cocktail of azo dyes, as does the *sindoor* or vermilion powder applied to the hair parting and sacred threads worn as religious amulets.[9] This has resulted in a situation where Indian consumers, both men and women, are exposed to various melanocytotoxic chemicals in close approximation to the skin. Our hot and humid weather further allows easy penetration of these chemicals through the compromised stratum corneum. Because of these factors, there have been increasing reports of chemical leucoderma in Indian patients in recent literature. Some well-defined situations where chemical leucoderma presents in recognizable patterns in our patients are discussed in the following sections.

## *Bindi* Leucoderma

**Figure 1:**
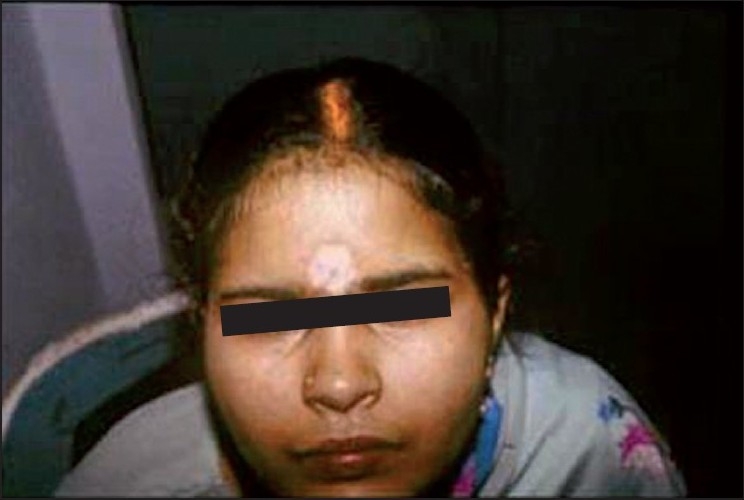
*Bindi* depigmentation

This depigmentation occurs in women at an extremely prominent site (center of the forehead) [Figure 1]. Moreover, the pressure to conform to religious and social norms makes many women continue to use the offending article even when the damage caused by it is clearly evident. The sticker type of *bindi* is the main cause, and the causative agent is PTBP present in high concentration in the glue.[7] This chemical is a known melanocytotoxic agent and depigmentation in workers exposed to it during its manufacture has been reported from Japan,[10] Holland,[1] Russia,[11] and the UK.[12] Among *bindi* users, it affects a small fraction, typically those women who wear it continuously day and night. The lag period between use and depigmentation is highly variable, ranging from a few weeks to a few years. An irritant dermatitis preceded the onset of depigmentation in almost three-quarters of women in a previous study.[7] On patch testing with the adhesive material, 5 of 15 patients showed positive reaction and three out of those developed depigmentation at the test site after 15–60 days. Upon laboratory testing, free PTBP in very high concentration (up to 80%) was found in various samples of the adhesive. Low concentrations of PTBP did not produce any positive reactions, ruling out sensitization in these patients. Although generalized vitiligo apparently triggered by occupational exposure to PTBP has been reported,[13] this appears to be relatively rare in our patients. Other common sources of exposure to PTBP are deodorants, spray perfumes, detergents, and household cleansers, all of which have been reported to cause chemical leucoderma in Indian patients.[9]

The treatment of *bindi* leucoderma hinges upon the early recognition of the condition (which is usually easy) and cessation of use of sticker *bindis* (which can be difficult). Orthodox Hindu women are strongly conditioned to wear a *bindi* at all times, especially in public. The alternative to a sticker *bindi* is a liquid red dye or *kumkum*, which is messy and difficult to use, apart from posing as yet an unknown risk from further damaging the skin from its own ingredients. While early stoppage of exposure can lead to slow spontaneous repigmentation, active treatment of *bindi* depigmentation is typically more difficult than vitiligo, since PTBP can permanently destroy the melanocytes within a few days of continuous use. However, topical corticosteroids and/or phototherapy are usually beneficial, with resistant cases requiring melanocyte transfer surgery.

## Synthetic Leather/Rubber Items Causing Leucoderma

**Figure 2:**
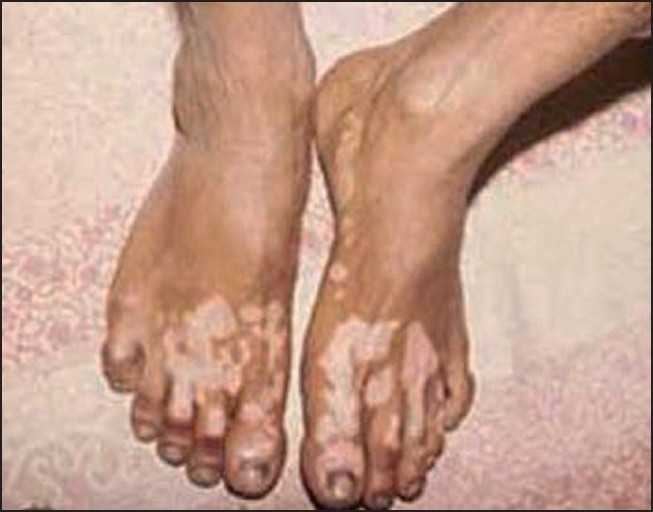
Footwear depigmentation

**Figure 3:**
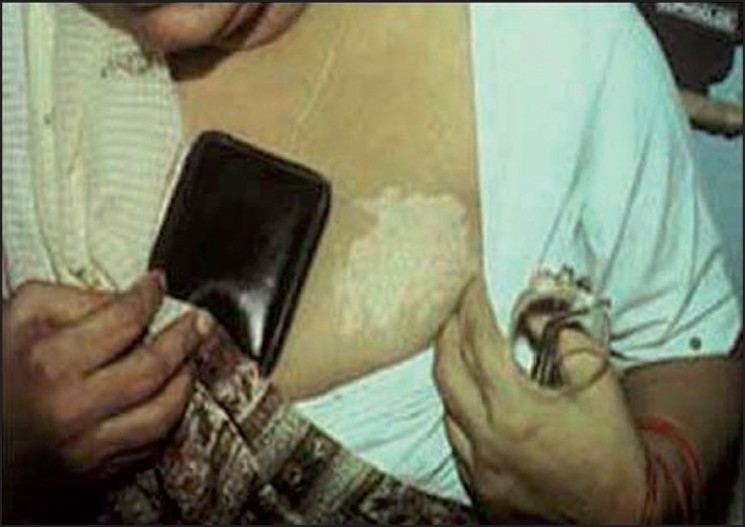
Wallet depigmentation

Common items from which synthetic leather-/rubber-related leucoderma has been reported are rain shoes, [Figure 2] rubber slippers, synthetic leather wallets (particularly when held directly against the skin, usually in women [Figure 3]), condoms, watch straps, hearing aid, and rubber gloves.[9,14,15] Almost all cases occur when the offending item is in tight apposition against the skin, and is kept there for prolonged periods leading to leaching of the offending chemical.

The common thread in depigmentation caused by these items of day-to-day use is monobenzyl ether of hydroquinone (MBH), also known as “Agerite Alba.” MBH is a rubber antioxidant and is well known to cause chemical leucoderma at sites of direct exposure, as well as distant sites on the body of predisposed individuals.[16] The depigmentation often starts as a cluster of guttate, confetti-like round macules that later coalesce to form large patches clinically indistinguishable from vitiligo. Usually, the depigmented patches conform closely to the shape of the offending article. An irritant dermatitis may precede its appearance in some cases. Although the earliest reports of chemical leucoderma to MBH from the United States[17] reported a high incidence of allergic sensitization, this has not been seen in Indian patients. Leucoderma can be reproduced at the patch test site with 20% MBH after a variable period, but not consistently.[18]

Other related compounds such as monomethyl ether of hydroquinone, pyrocatechol, and hydroquinone have also been reported to cause chemical leucoderma, but these are rarer culprits than MBH.[18] Commercial bleaching creams containing –5% hydroquinone have rarely been reported to cause chemical leucoderma as have photographic developing solutions containing 0.06– 7% hydroquinone.[19] Mercaptobenzthiazole (MBT), a rubber accelerator, has been reported as the cause in a patient who developed depigmentation on the penis after using latex condoms. This patient was also allergic to MBT and Latex condom.[15]

Reports of depigmentation to MBH and related chemicals from India[9,14,15] are usually to household objects, whereas western reports are usually from an industrial setting.[20] This is also true of other chemicals producing leucoderma. The poorly regulated nature of the small- and medium-scale manufacturing industries in India can probably account for this difference. For example, Taylor *et al*.[21] have reported that the US rubber industry has stopped using MBH in processing for several years now, whereas Indian-made rubber items still contain MBH. Similarly, the very high concentrations of free PTBP found in some Indian sticker *bindis*[7] are not found in western society outside of factories producing the chemical itself. Another cause of paucity of Indian reports of occupational depigmentation is probably the under-developed nature of occupational medicine in general and occupational dermatology in particular.

Treatment is on similar lines as *bindi* leucoderma, except that exquisite photosensitivity has been seen many patients with footwear dermatitis by one of the authors (AKB), making psoralen and ultraviolet A (PUVA) or any other form of phototherapy difficult.

## Hair Dye Depigmentation

**Figure 4:**
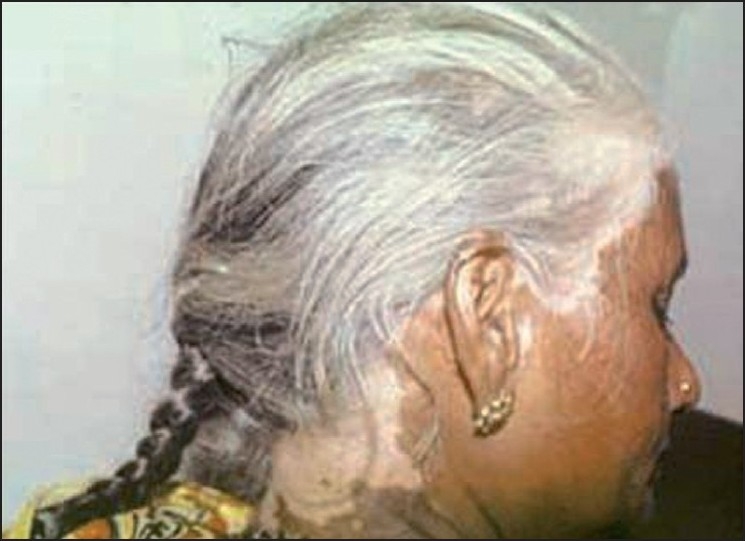
Hair dye depigmentation

Ever since Taylor *et al*.[22] described four cases of leucoderma caused by hair colors, there has been a stream of similar reports, including those from India.[23] In many cases, the depigmentation is preceded by a dermatitis, but this is not essential [Figure 4]. In most of these cases, the cause of selective melanocytotoxicity is PPD, and depigmentation at patch test sites has also been reported after a few months of testing.[23] These reports are especially alarming for us because the Indian hair dye market is dominated by small poorly regulated brands that have been shown to contain extremely high concentrations of PPD.[23] Since PPD is a very common sensitizer, the possibility of it triggering the onset of vitiligo in other areas in genetically susceptible individuals is also significant, as was seen in an earlier report.[9] Other items from which chemical depigmentation caused by PPD has been reported are black socks and shoes.[9]

Repigmentation of hair dye leucoderma with narrow band ultraviolet B(NB UVB) phototherapy has been reported recently.[24] At least theoretically, the chances of improvement with phototherapy in dense hair bearing areas are better since the melanocyte reservoir in the hair follicles is likely to be more abundant in other areas with lower hair density.

## *Alta* and Amulet String Leucoderma

**Figure 5:**
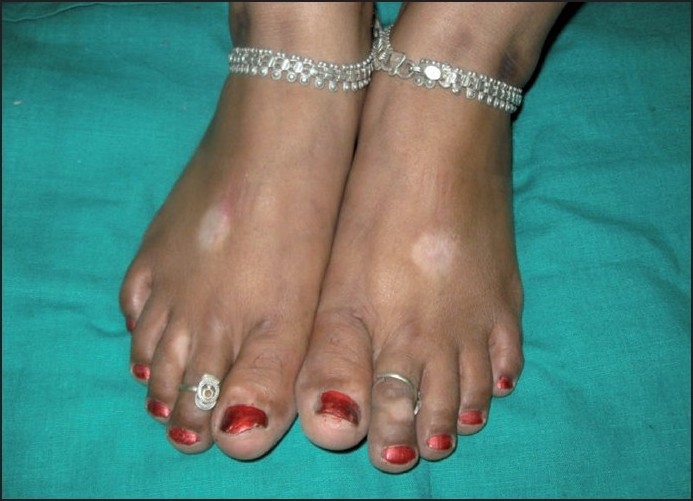
*Alta* depigmentation

This entity is almost entirely confined to India and presumably its neighbors, where *alta* [Figure 5] and colored threads are worn for socio-religious reasons.[9,25] The depigmentation in these cases is caused by azo dyes, which are present in several items of everyday use, including colored clothes. Other items that have been reported to cause azo dye-related depigmentation are lipsticks, lip liners, eyeliners, and *faux* fur from soft toys.[9] The bulk of investigation on this entity has focused on *alta*-related cases. This scarlet red solution is commonly applied by women to their feet on festive occasions and has been found to contain mainly two dyes, namely Crocein Scarlet MOO and Rhodamine B (an inert dye) as well as Solvent Yellow 3, possibly a contaminant.[26] Crocein Scarlet MOO and Solvent Yellow 3 are azo dyes. These dyes easily undergo oxidation in the air, which is further catalyzed by sunlight. This produces several complex compounds, which may contribute to the melanocytotoxicity of these dyes.[27] There is significant structural homology between PPD and azo dyes, and cross-reactions are well known. Crocein Scarlet MOO and Solvent Yellow 3 have been shown to be capable of causing depigmentation.[28]

## Miscellaneous Chemical Leucodermas

Mathias *et al*.[29] reported perioral depigmentation in a patient using cinnamic aldehyde- containing toothpaste in whom the patch test sites also depigmented. Recently, toothpaste-induced chemical leucoderma has also been reported in some patients from India,[9] although it is not clear whether these patients were patch tested. Since the oral mucosa and lips are common sites for occurrence of vitiligo, these patients need to be carefully examined for distant lesions before diagnosing chemical leucoderma.

Recently, ophthalmologists from Pondicherry[30] reported a patient who presented with an allergic reaction to olopatadine eye drops that was rapidly followed by periocular depigmentation. Several other eye preparations have also been reported to cause similar leucoderma, namely physostigmine, diisopropyl fluorophosphates, thiotepa, and guanonitrofuracin.[4]

## The Mechanism of Chemical Leucoderma and Implication on Treatment

The exact mechanism by which these chemicals cause damage to the melanocytes is still unknown, but laboratory investigations have shown that some azo dyes have the capacity to bind with human DNA, which can cause direct cell death by apoptosis.[31] It has also been demonstrated that 4-tertiary butyl phenol, which is a potent inducer of chemical leucoderma, can induce apoptosis in melanocytes.[32] There is also evidence that azo dyes, by virtue of structural similarity to tyrosine, can directly inhibit melanogenesis by competing with that amino acid for binding with tyrosinase enzyme.[27] It is postulated that the hydroxylation products that are formed when phenolic compounds react with tyrosinase can cause oxidative damage to the melanocyte. Manga *et al*.[33] have recently shown that another melanocyte-specific enzyme, tyrosinase-related protein-1 mediates the melanocyte damage caused by phenolic compounds, presumably by producing intermediates that overwhelm the cellular antioxidant systems.

From the previous discussion, it is clear that chemical leucoderma can result both from an inhibition of melanin production in otherwise viable melanocytes and from melanocyte cell death. Obviously, the treatment outcome would be very different in both scenarios. Although it is not clear whether melanocyte destruction is dependent on the duration of exposure to these chemicals, time dependency has been shown in guinea pigs exposed to monoethyl ether of hydroquinone.[34]

In conclusion, chemical leucoderma presents in several identifiable patterns in Indian patients and the causative agent can be identified and instructions for its avoidance given in most cases. Due to our lax product safety laws, a lot of potentially melanocytotoxic chemicals come in contact with our skin everyday. Therefore, it is important for every dermatologist to acquaint him/herself with this condition and prevent the devastating sequelae of permanent melanocyte destruction in our patients.
